# Continuous wireless postoperative monitoring using wearable devices: further device innovation is needed

**DOI:** 10.1186/s13054-021-03805-0

**Published:** 2021-11-15

**Authors:** William Xu, Armen A. Gharibans, Ian P. Bissett, Gregory O’Grady, Cameron I. Wells, Carlos Areia, Christopher Biggs, Mauro Santos, Neal Thurley, Stephen Gerry, Lionel Tarassenko, Peter Watkinson, Sarah Vollam

**Affiliations:** 1 Department of Surgery, University of Auckland, Building 507, 22-30 Park Avenue, Grafton, Auckland, 1145 New Zealand; 2 Auckland Bioengineering Institute, University of Auckland, Auckland, New Zealand

To the editor,

We read with great interest the recent article by Areia et al. which highlights the current lack of evidence surrounding the use of wearable monitoring systems to detect deterioration in hospitalised patients.

We agree with the authors’ conclusion that the variety of designs, populations, outcomes, and devices “makes it difficult to reach a definitive conclusion”. We also agree that this is an emerging area needing further research.

In this meta-analysis, three small randomised controlled trials (RCTs) were included. These RCTS utilised two different devices, the SensiumVitals (The Surgical Company, UK), and the Isansys Lifetouch (Isansys Lifecare, UK) devices. The limited precision of both devices has been noted previously [1, 2]. Other clinical validation studies of wearable monitoring devices have also demonstrated limited device precision in the postoperative setting [3]. Therefore, any assessment of the ability for devices to detect early deterioration is heavily confounded by inherent limitations of the devices. The possibility for wireless continuous monitoring cannot be realised until the devices used are sufficiently accurate and precise.

Despite the breadth of devices in this field, most wireless continuous monitoring devices are still in their clinical validation phases [4]. The most immediate challenge in this field is the need for more accurate devices, which needs to be solved through further innovation. Large, well-powered RCTs should not be conducted until robust clinical validation studies have demonstrated adequate device accuracy and precision. Validation studies should also consider the use of continuous monitors as a reference standard rather than nurse-measured observations which are limited in both their frequency and potential accuracy.

Wireless continuous vital signs monitoring likely has benefits that are yet to be apparent. Undetected hypotension, hypoxemia and respiratory depression in the postoperative patient is common and often prolonged; delayed detection of these physiological changes may be a significant contributor to ‘failure-to-rescue’ and postoperative death [5]. Systems that allow for better detection of patient deterioration have the potential to significantly improve patient care, and rigorous work should be done to explore avenues for improvement. Accurate artefact rejection, better signal processing, and novel sensor technology all may improve device accuracy and utility.

The work of Areia et al. has highlighted the need for further innovations in this space. Device improvements and clinical validation work will shed light on the true value of continuous postoperative monitoring and, most importantly, help confirm if this technology can improve patient outcomes.

## Data Availability

Not applicable.
