# Supplementary material for: Continuous wireless postoperative monitoring using wearable devices: further device innovation is needed
Source: Crit Care. 2021 Nov 15;25:394. doi: 10.1186/s13054-021-03805-0 (PMC8594168; doi:10.1186/s13054-021-03805-0)
Supplement: Related texts

# Reply: Authors’ response

**Authors:** Carlos Areia, Christopher Biggs, Mauro Santos, Neal Thurley, Stephen Gerry, Lionel Tarassenko, Peter Watkinson, Sarah Vollam

We would like to thank Xu et al. for their interest in our recent manuscript.

We agree most devices are still in their feasibility and clinical validation stage, with limited evidence on their clinical and cost effectiveness, highlighted in our review and previous research [4].

It is important to consider that the goal of this emerging technology should be to support, and not replace, clinical vital sign monitoring practice, offering another layer of protection between traditional continuous monitoring and manual intermittent observations. Furthermore, although accuracy and reliability of wearable devices are a barrier, most studies have been conducted in healthy volunteers and in comparison with clinical and/or gold standard for continuous measurement [4]; with even less evidence comparing these devices with the standard intermittent manual measurements, the common practice in general wards.

We agree with our colleagues these new monitoring systems should not be used as the “stand-alone” method of vital sign measurement, due to current accuracy and precision limitations. However, well-powered and well-designed clinical trials, including careful implementation of these new systems, might still be of benefit to support the development and innovation of these technologies, by testing its impact in clinical care as complementary, and not a substitute, to standard practice [6].
